# Pregnant women adherence level to antenatal care visit and its effect on perinatal outcome among mothers in Tigray Public Health institutions, 2017: cohort study

**DOI:** 10.1186/s13104-018-3987-0

**Published:** 2018-12-07

**Authors:** Abera Haftu, Hadgay Hagos, Mhiret-AB Mehari, Brhane G/her

**Affiliations:** Department of Midwifery, Mekele University College of Health Sciences, Tigray, Ethiopia

**Keywords:** Pregnant women, Adherence, Antenatal care

## Abstract

**Objective:**

To assess pregnant women adherence level to antenatal care visit and its effect on perinatal outcome among mothers in Tigray Public Health institutions, 2017.

**Results:**

The overall adherence level of the women towards to antenatal care visit was 49.9% and incidence of PPH, still birth, early neonatal death, late neonatal death and low birth weight complication was 4.3%, 2.3%, 2.7%, 1.9% and 7.5% respectively. PPH, preterm labor, early neonatal death and LBW complication was reduced by 81.2%, 52%, 61% and 46% respectively among women’s with complete adherence to ANC visit.

## Introduction

Complications that happen during pregnancy and childbirth are the most leading causes of maternal mortality and morbidity among women whose age ranges from 15 to 49 in developing countries [1]. Annually around 287,000 women die secondary to pregnancy related cause in the globe, among this figure 99% of the maternal death is from underdeveloped countries [2]. Ethiopia is among the leading countries with high maternal mortality and morbidity from the developing countries [3]. In developing countries almost all pregnant women’s receive antenatal care at least once, but in sub-Saharan countries the report is around 68% where women’s take antenatal care (ANC) services at least ones and majority of them visit the health institutions at third visit [4, 5].

Most research finding showed that most of maternal and neonatal deaths are preventable; one the strategic and important key step for reducing of maternal related mortality and morbidity is antenatal care directly by detecting and treating of complications in earlier period starting from the onset of pregnancy till delivery [6]. The timing of starting first ANC and total number of ANC visits that pregnant women receive and not attending the recommend ANC services may lead to adverse perinatal outcomes [7].

Ethiopian Demographic Health Survey (EDHS) 2016 showed that national ANC service coverage is around 64%, even if the total number of ANC visit is good, starting ANC follow up in the earlier second trimester is low in magnitude. Research results of late ANC service booking from Addis Ababa, Metekel, Hadiya, Ambo and Gondar was 59.8%, 55.1%, 68.2%, 86.8% and 64.9% respectively [8–12]. In the current situation Ethiopia deliveries three tiered health care system; this is characterized by district health system, health centers and health posts which are connected to each other by referral system [13]. The need of ANC is taken as basic rights of all pregnant women’s to keep safe their infants, the high maternal and neonatal mortality in Ethiopia is the result of poor utilization of ANC [14, 15]. The primary target of ANC is to detect problem, treat on time and prevention of complications by health care provision, despite of this illiteracy and low socio economic status contribute to poor ANC adherence. There have many studies which showed positive effect of ANC on perinatal outcome including reducing risk of postpartum hemorrhage (PPH), low birth weight, preterm birth and perinatal death. World health organization (WHO) recommended for all pregnant women to have four consecutive ANC visits for low risk pregnant women’s [16–25].

## Main text

### Methods and materials

#### Study area and period

The study will be conducted in Tigray Public Health institutions. Tigray is located in Northern part of Ethiopia and around 783 km away from the capital city Addis Ababa. Around 5.5 million people are found in this region (census 2007). The region is the owner of 216 health centers, 15 General Hospitals and 2 Referral Hospitals. Among the selected zones (southern, Mekele & southeastern zones) there are about 61 health centers, 5 primary Hospitals 1 Referral Hospital and 6 General Hospitals. The study was conducted from July 1, 2017 to August 2018.

#### Study design

Prospective cohort design was employed.

#### Source population

All women’s who gave birth in Tigray Public Health institutions.

*Study population* Women’s who fulfill the criteria and selected in the study period.

*Exposed group* Are mothers coming to the health facility for delivery services where their ANC visit was complete.

*Non-exposed group* Are mothers coming to the health facility for delivery services where their ANC visit was incomplete.

#### Eligibility criteria

*Inclusion criteria* All women coming for delivery services in the public health facilities.

*Exclusion criteria* Women who has known medical illness (hypertension, cardiac disease, DM, malaria, liver disease).

#### Sample size determination

Sample size was calculated using double population proportion formula for cohort study considering the following assumptions:

- CL = 95%.
- Power-80%.
- A one-to-one ratio of exposure to non-exposure.
- Since there is no any documented evidence in the setting, it is assumed that the complication rate will be twice as high amongst the exposed group (complete adherence) as compared to unexposed group (incomplete adherence).
- By taking prevalence of pregnancy complication (PIH/preeclampsia–eclampsia) among the mothers with complete adherence to be 5.1% from previous study in Ghana [11].
  $$ n_{1} = \frac{{\left[ {Z_{\alpha /2} \sqrt {\left( {1 + \frac{1}{r}} \right)P(1 - P)} - Z_{{\beta \sqrt {P_{1} (1 - P_{1} ) + \frac{{P_{2} (1 - P_{2} )}}{r}} }} } \right]}}{{(P_{1} - P_{2} )^{2} }}^{2} $$
- The final total sample size is 928.
- 464 participants in each group.

#### Sampling technique

Systematic random sampling was used for this population and the sample was distributed to each facility based on proportional allocation in correspondence of delivery services. Women who have full visits were considered as exposed group where as those with incomplete follow up will be considered as non exposed group. Exposed and non exposed mothers who fulfill the inclusion criteria were enrolled to the cohort and were followed until the end of post partum period. Among the seven zones of the region 40% of them were selected by simple random selection technique. In the selected zones there are about 73 health facilities, by using simple lottery method 20 of them will be selected. The sample size will be distributed to each selected health facility by probability proportion to size (PPS) according to their ANC flow rate.

#### Data collection technique and process

Women’s who come for delivery services in the public health institutions who met the criteria for the cohort study were enrolled and followed till the end of the postpartum period. After reviewing the women’s document based on their ANC frequency they were recruited to exposed and non exposed groups, those with complete adherence ANC visits were considered as exposed groups and those incomplete ANC visits were considered as non exposed groups. Questioner was prepared from different literatures and WHO recommendations for pregnancy, delivery and post delivery continuum of care. There were about 20 BSC Midwives data collectors one data collector per each health facility. Three day data training was given to the data collectors and frequent supervision was made to each health facilities at 1 week interval. The follow up was at respective health institutions, but for those who were unable to attend the follow up health facility required information was collected by telephone. In order to ensure adherence of the follow up the community was mobilized by health extension workers

Data was entered by Epi data version 3.1 software first then exported to SPSS version 20 software for analysis purpose. Descriptive analysis was presented using mean and proportions. Tables, figures and text were used for data presentation. Determinants of maternal and neonatal complications, as well as the effect of complete adherence on pregnancy outcomes was estimated and expressed as relative risks (RRs) with their 95% confidence intervals (CI). Binary logistic regression run to see the association between variables. Significance was declared at *p* value < 0.05.

#### Data quality assurance

Standardized English version measuring questionnaire was adapted and it was translated into Tigrigna (local language) by experts. The questionnaire was reviewed by senior researchers and comments were incorporated for internal validity. In addition it was pre-tested on 10% of the calculated sample size. Data collectors and supervisors were trained for 3 days on the tools and process of data collection. Five percent of the collected data was checked by the supervisor for completeness and finally the investigators will monitor the overall quality of data collection.

#### Variables

*Dependent variable* Perinatal outcome.

*Independent variable* Socio-demographic factors (age, educational level, marital status and employment status).

Maternal factors (parity, trimester at first antenatal care visit, previous pregnancy history and number of times antenatal clinic was attended during pregnancy).

Neonatal factors (mode of delivery, duration of delivery, place of birth).

#### Operational definitions

*Complete adherence* women’s who attend the ANC visit four and above.

*Incomplete adherence* women who had attended ANC visit less than or equal to three time.

### Results

A total of 1103 women were recruited and 100% of them were followed up to 6 weeks post-partum from December 2017 to July 2018. The number of women’s enrolled to the exposed and non exposed groups were above the minimum size set during the proposal writing, this is done to meet the minimum number of participants to each groups in the selected health institutions.

#### Participants’ baseline characteristics

The age of the participants in mean and standard deviation were 26.4 (5.2) years. 45.3% of the participants’ age was 19–25 years. Most of the participants’ educational level were secondary school and above which makes 42.3% from the total. More than half of the participants (71.8%) were from urban residence and married individuals take the highest proportions (92.7%) from the total participants (see Table 1).

**Table 1:** Socio demographic characteristics of study participants

Variable	Frequency	Percent
Age		
≤ 18	33	3
19–25	500	45.3
26–30	356	32.3
31–35	156	14.1
≥ 36	58	5.3
Residence		
Urban	729	71.8
Rural	311	28.2
Marital status		
Married	1023	92.7
Single	52	4.7
Divorced	23	2.1
Widowed	5	0.5
Religion		
Orthodox	1011	91.7
Muslim	79	7.2
Catholic	5	0.5
Protestant	8	0.7
Educational level		
No education	283	25.7
Read and write	99	9
Primary school	254	23
Secondary school and above	467	42.3
Occupation		
House wife	729	66.1
Government employee	157	14.2
Nongovernmental employee	34	3.1
Private organization	147	13.3
Daily laborer	17	1.5
Other	19	1.7
Ethnicity		
Tigray	1088	98.6
Amahara	13	1.2
Other	2	0.2
Monthly income (birr)		
≤ 500		4.5
501–1500	64	5.8
1501–3000	310	28.1
3001–5000	351	31.8
5001–1000	203	18.4
≥ 10,001	153	13.9

#### Incidence of maternal and neonatal complications

Overall the incidence of postpartum hemorrhage is 4.8% of which 1.6% and 6.9% are from women’s with complete and incomplete adherence respectively. Still birth and asphyxia were 2.3% and 10.3% consecutively (see Table 2).

**Table 2:** Incidence of the maternal and neonatal complications among the two groups (adhered versus non adhered)

Complication	Incidence (%) N = 1103	Complete adherence N = 550	Incomplete adherence N = 553
Maternal			
PPH	47 (4.3)	9 (1.6)	38 (6.9)
Maternal infection	90 (8.2)	34 (6.2)	56 (10.1)
Admitted to ICU	28 (2.5)	13 (2.4)	15 (2.7)
Incomplete postnatal visit	452 (41)	170 (30.9)	282 (50.9)
Neonatal			
Still birth	25 (2.3)	5 (0.9)	20 (3.6)
Neonatal sepsis	111 (10.1)	55 (10)	56 (10.1)
Early neonatal death	30 (2.7)	8 (1.5)	22 (4)
Late neonatal death	21 (1.9)	6 (1.1)	15 (2.7)
Asphyxia	114 (10.3)	49 (8.9)	65 (11.8)
Low birth weight	83 (7.5)	31 (5.6)	52 (9.4)

#### Effect of complete adherence on risk of pregnancy complications

Postpartum hemorrhage complication was reduced by 81.2% among women’s with complete adherence to antenatal care visit [ARR = CI 95% = 0.188 (0.088–0.404)]. Early neonatal death was reduced by 61.3% [ARR = CI 95% = 0.387 (0.162–0.928)] and low birth weight was reduced by 46.5% [ARR = CI 95% = 0.535 (0.326–0.878)] among women’s with complete adherence to antenatal visit (see Table 3).

**Table 3:** Multivariate analysis of complete adherence’s effect on maternal and neonatal complications

Complication	Crude RR 95% CI	p-value	Adjusted RR 95% CI	p-value
PPH	0.225 (0.108–0.471)	0.000	0.188 (0.088–0.404)	*0.000*
Preterm labor	0.425 (0.267–0.673)	0.000	0.476 (0.290–0.783)	*0.003*
Maternal sepsis	1.73 (1.110–2.696)	0.015	1.610 (0.986–2.630)	0.057
Neonatal sepsis	1.026 (0.693–1.520)	0.896	0.833 (0.544–1.275)	0.400
Early neonatal death	0.356 (0.157–0.807)	0.013	0.387 (0.162–0.928)	*0.033*
Late neonatal death	0.396 (0.152–1.027)	0.057	0.380 (0.138–1.052)	0.060
Low birth weight	0.575 (0.363–0.913)	0.019	0.535 (0.326–0.878)	*0.013*
Delivery complication	1.173 (0.922–1.492)	0.193	1.185 (0.912–1.541)	0.204
Neonatal complication	1.008 (0.778–1.305)	0.953	0.920 (0.695–1.217)	0.558

### Discussion

Many studies have shown the positive effect of antenatal care services on perinatal outcome, so giving emphasis to determine the gap with women adherence to antenatal visit and its effect on perinatal outcome is timely and significant as woman with single visit and four visits will not have similar complications.

Overall the women’s adherence to complete visit to antenatal care is 49.9% and the follow up till postpartum period was 100% complete in our study where as research findings from Addis Ababa, Metekel, Hadya Zone, Ambo and Gonder show that the prevalence of ANC service booking was 59.8%, 55.1%, 68.2%, 86.8% and 64.9%, respectively [8–12]. This variation could due to the geographical location and the documentation system and manly the study design in general as they use the snap shoot kind of study where as in this study the focus was on the full course of the antenatal care.

In this study women’s who attend at least single antenatal care was much lower than other studies (7%) which was done in Nepal, Pakistan, Bangladesh and Indian which was 28%, 28%, 33% and 60% respectively [26]. This variation again could be due to the educational level and economic status of the participants.

In this study the incidence of low birth weight was 7.5% which is a little bit higher than a study done in Nigeria which was 4.8% among women’s with greater than or equal to four visits [27] and this variation could be due the nutritional consumption and geographical location.

Most studies from what we had searched showed that prevalence and determinant factor for ANC utilization, less emphasis was given to women’s level of adherence to ANC visits and its effect on perinatal outcome. The incidence of developing postpartum hemorrhage among women’s with complete adherence to antenatal care visit was about 1.6% where as in women’s with incomplete adherence was 6.9% and incidence of adherence to postnatal visit was 30.9% and 50.9% among women’s with complete and incomplete adherence respectively.

This study shows that incidence of neonatal complication is higher among the women’s neonate with incomplete adherence to antenatal visit. Incidence of still birth among women’s with incomplete adherence is four fold of the women’s with complete adherence to antenatal visit which is 3.6 and 0.9% respectively. The incidence of early neonatal death and late neonatal death among women’s with complete adherence to antenatal visit was almost similar which is 1.5 and 1.1% respectively where as among the women’s with incomplete adherence to antenatal care visit the incidence of the early neonatal death is higher than the late neonatal death which is 4 and 2.7% consecutively and this could be due to the vulnerability of the neonate to many things in the earlier period than late after they customize the environment. The incidence of low birth weight among the non exposed group was almost twice of the exposed group (9.4%:5.6%) and this might be probably due to the counseling regarding nutritional methods during antenatal visit.

## Limitation

- Conducted at different sites in which it was difficult to supervise timely.
- Geographical location was one factor during the follow up.
- Delayed to meet them during follow up b/c of the method of communication.

## Abbreviations

ANC: antenatal care
EDHS: Ethiopian Demographic Health Survey
PPH: postpartum haemorrhage
WHO: World Health Organization
